# Low Dose of Carbendazim and Tebuconazole: Accumulation in Tissues and Effects on Hepatic Oxidative Stress in Mice

**DOI:** 10.3390/toxics11040326

**Published:** 2023-03-30

**Authors:** Xiaoran Ma, Xin Chen, Haonan Hou, Donghui Liu, Xueke Liu, Peng Wang, Zhiqiang Zhou

**Affiliations:** Department of Applied Chemistry, College of Science, China Agricultural University, Beijing 100193, China

**Keywords:** carbendazim, tebuconazole, residue, oxidative stress, acceptable daily intake

## Abstract

As two commonly used fungicides, carbendazim and tebuconazole are widely found in the environment and in foods. Studies have reported that these fungicides can induce hepatic oxidative stress and other health risks. Nevertheless, the influences of exposure to carbendazim and tebuconazole at their acceptable daily intake (ADI) doses on hepatic oxidative stress, and the residual distributions in mice remain unclear. To fill these gaps, ICR (CD-1) mice were exposed to carbendazim and tebuconazole at their ADI doses by oral administration for 4 weeks in this study. The results showed that tebuconazole accumulated primarily in the epididymal fat of mice (16.84 μg/kg), whereas no significant residues of carbendazim in the tissues were observed. In addition, exposure to ADI doses of tebuconazole significantly reduced liver coefficients and induced hepatic oxidative stress in mice, including elevating the levels of glutathione and malonaldehyde. However, no significant impacts were observed on the hepatic redox homeostasis in mice after exposure to carbendazim at its ADI dose. The results could be helpful for understanding the exposure risks of carbendazim and tebuconazole in terms of low doses and long term.

## 1. Introduction

Carbendazim (methyl 1H-benzimidazol-2-ylcarbamate) is a broad-spectrum benzimidazole fungicide that controls various fungal pathogens. It is also used as a preservative in the papermaking, paint, leather industries, and fruits [1,2]. Studies have shown that carbendazim can have potential impacts on non-target organisms. Carbendazim can trigger oxidative stress and apoptosis in zebrafish larva development [3]. Exposure to sublethal doses of carbendazim can induce brain oxidative stress and lead to the inhibition of acetylcholinesterase (AChE) in juvenile African catfish [4]. It has been reported that carbendazim can induce neurotoxicity through the disorder of redox homeostasis and activation of the NF-κB signaling pathway in male Wistar rats [5]. Similarly, exposure to carbendazim can lead to hepatorenal damage via oxidative stress and programmed cell death in a dose-dependent manner in rats [6]. Chronic exposure to carbendazim can induce lipid metabolic disruption and intestinal microbiota dysbiosis, and further elicit inflammatory responses in multiple tissues in mice [7]. As an endocrine disruption chemical (EDC) [8,9,10], carbendazim can lead to reproductive toxicity by damaging steroidogenic enzymes and enhance lipid peroxidation in the Leydig cells in rats [11]. Carbendazim at 0.1 mg/kg, can impair spermatogenesis through the estrogen receptor pathways in male ICR mice [12].

As a triazole fungicide with broad-spectrum antifungal activity, tebuconazole (α-(2-(4-chlorophenyl)ethyl)-α-(1,1-dimethylethyl)-1H-1,2,4-triazole-1-ethanol) is widely used for the control of various fungal pathogens in fruits, vegetables, and cereals [13,14]. With its high usage and long half-life, tebuconazole is widely spread in the natural environment [15], which leads to substantial risks for non-target organisms [16,17]. Studies have shown that tebuconazole can inhibit brain AChE activity and reduce the exploratory behavior in zebrafish larvae [18]. Transcriptomic results have shown that tebuconazole can pose potential neurotoxicity, immunotoxicity, and carcinogenicity in earthworms [19]. Tebuconazole can induce cytotoxic and genotoxic effects in intestinal cells (HCT116 cells) [20] and stimulate apoptosis in placental trophoblast (HTR-8) cells [21]. Furthermore, tebuconazole administration can induce oxidative stress in the liver of fruit-eating bats [22], zebrafish [23], and male C57BL/6 mice [16]. Sub-chronic exposure to tebuconazole can cause obvious renal toxicity by damaging renal tissue structure, inducing renal oxidative stress, and triggering apoptotic signals in the kidneys of male Wistar rats [24]. In addition, exposure to tebuconazole can induce colonic inflammation in mice by causing inflammatory cell infiltration in colon tissue, activating the expression of inflammation-related genes, and disrupting the intestinal barrier function [25].

Although there have been numerous studies showing that carbendazim and tebuconazole pose a variety of health risks to non-target organisms, the exposure doses of these two fungicides in the studies were generally much higher than the environmental residue concentrations and government-established residue limits (such as maximum residue limits, MRLs) for them on agricultural products. The health risks of exposure to safe low doses (e.g., doses of acceptable daily intake, ADI) of carbendazim and tebuconazole are still unknown. The ADI of a pesticide is the daily intake that presents no appreciable risk to consumer health during the entire lifetime based on the known animal studies at the time of the evaluation of the pesticide by the Joint FAO/WHO Meeting of Pesticide Residues (JMPR). In addition, it is generally believed that the liver is the primary target organ of pesticide toxicity. As xenobiotic pollutants, carbendazim and tebuconazole enter the mouse body and migrate to the intestinal tract through the digestive tract. Parts of these pesticides are excreted through feces and urine, while other parts are transferred to the liver to be metabolized by the hepatic microsomal enzyme system. Carbendazim and tebuconazole might induce hepatic toxicity during the process. Therefore, we assessed the residues and hepatic oxidative stress in mice exposed to carbendazim and tebuconazole at their ADIs. To analyze whether the fungicides would cause residues and induce hepatic oxidative stress in a dose-dependent manner, the influences of exposure to carbendazim and tebuconazole at their 100-fold ADI doses were also investigated.

In this study, the extraction and analysis methods of carbendazim and tebuconazole in mouse organs were established. Furthermore, the accumulations of the two fungicides in the major organs of mice following 4-week exposure at their ADI doses were investigated. In addition, the influences of individual exposure to carbendazim and tebuconazole at their ADI doses on hepatic oxidative stress in mice were assessed. This study could provide data on the distribution characteristics after exposure to carbendazim and tebuconazole at safe low doses and on the adverse effects on the hepatic redox homeostasis.

## 2. Materials and Methods

### 2.1. Chemicals

Carbendazim (CAR) (CAS No. 10605-21-7, 98.2% purity) and tebuconazole (TEB) (CAS No. 107534-96-3, 97% purity) were obtained from the Institute for the Control of Agrochemicals, Ministry of Agriculture of China (Beijing, China). Corn oil was purchased from Shanghai Aladdin Biochemical Technology Co., Ltd. (Shanghai, China). All other chemicals and solvents were purchased from commercial sources.

### 2.2. Animals and Experimental Design

Four-week-old male CD-1 (ICR) mice were purchased from SiPeiFu (Beijing, China) Biotechnology Co., Ltd. (Beijing, China). The mice were maintained in a specific pathogen-free (SPF) environment at 23 ± 2 °C in 12/12 h light/dark cycles. Following one week of acclimation, the male mice were randomly divided into three groups (*n* = 7 for each group). The first group was administered with carbendazim by daily gavage at its acceptable daily intake (ADI) dose (0.03 mg/kg b.w. from JMPR Report 1995 [26], dissolved in corn oil), and was assigned as the CAR group The second group was administered with tebuconazole by daily gavage at its ADI dose (0.03 mg/kg b.w. from JMPR Report 2016 [27], dissolved in corn oil), and was assigned as the TEB group. The third group was administered with only corn oil by daily gavage as control, and was assigned as the CON group. To investigate the relationship between exposure dose and pesticide accumulation in mice, there were additional seven mice who were administered carbendazim and tebuconazole at their 100-fold ADI doses (3 mg/kg b.w., dissolved in corn oil) daily by gavage, and this group was assigned as the MIX group. Following 4 weeks of treatment, the mice were euthanized. The liver, kidney, spleen, epididymal fat, testis, brain, and heart were collected and snap-frozen immediately in liquid nitrogen, and then stored at −80 °C until analysis.

### 2.3. Biochemical Analysis

The mouse liver samples (100 mg) from each group were homogenized with 900 mL of saline solution in an ice bath for 60 s, and then centrifuged at 4000 rpm for 5 min at 4 °C. The supernatant was collected for the following measurements. The levels of total protein (TP), malondialdehyde (MDA), reduced glutathione (GSH), hydrogen peroxide (H_2_O_2_), and the activities of superoxide dismutase (SOD) were measured using commercial assay kits (Nanjing Jiancheng Bioengineering Institute, Jiangsu, China) according to the manufacturer’s protocols. In brief, the protein concentration was detected through the bicinchoninic acid (BCA) method, through which protein can reduce Cu^2+^ to Cu^+^ levels and change the color of the working reagent under alkaline conditions. Specifically, we added 250 μL of the working reagent into 10 μL of the liver homogenate, we mixed the sample gently, and then incubated it at 37 °C for 30 min. Then, we measured the absorbance (A) at 562 nm. The concentration of protein was calculated using Equation (1).

(1) $$\mathrm{protein}\;\mathrm{concentration}=\frac{A_{\mathrm{test}}-A_{\mathrm{blank}}}{A_{\mathrm{standard}}-A_{\mathrm{blank}}}$$

The level of MDA was tested using the thiobarbituric acid (TBA) method, in which MDA would condense with TBA and formed a red product. Following the instructions, 100 μL of reagent 1 was added to 100 μL of the liver homogenate and mixed evenly. Then, we added 3 mL of reagent 2 and 1 mL of reagent 3 to the mixture, and incubated it at 95 °C for 40 min. Then, the mixture was cooled by flowing water and centrifuged at 3500 rpm for 10 min. The absorbance of the supernatant was measured at 532 nm, and the concentration (C) of MDA was calculated using Equation (2).

(2) $$\mathrm{MDA}\;\mathrm{concentration}=\frac{A_{\mathrm{test}}-A_{\mathrm{blank}}}{A_{\mathrm{standard}}-A_{\mathrm{blank}}}\times C_{\mathrm{standard}}$$

The GSH content was measured by reacting the content with 5,5′-dithiobis-(2-nitrobenzoic acid) (DTNB). We added 100 μL of reagent 2 and 25 μL of reagent 3 to 100 μL of the liver homogenate. We mixed the sample and left it for 5 min. Then, we measured the absorbance at 405 nm, and the concentration of GSH was calculated using Equation (3).

(3) $$\mathrm{GSH}\;\mathrm{concentration}=\frac{A_{\mathrm{test}}-A_{\mathrm{blank}}}{A_{\mathrm{standard}}-A_{\mathrm{blank}}}\times C_{\mathrm{standard}}\times {\mathrm{reaction}\;\mathrm{coefficient}÷C}_{\mathrm{protein}}$$

The level of H_2_O_2_ was tested using the molybdic acid method. According to the manufacturer’s protocols, we added 1 mL of reagent 1 and 1 mL of reagent 2 to 100 μL of the liver homogenate. We mixed the sample evenly, and measured the absorbance at 405 nm. The level of H_2_O_2_ was calculated using Equation (4).

(4) $$H_{2}O_{2}\;\mathrm{concentration}=\frac{A_{\mathrm{test}}-A_{\mathrm{blank}}}{A_{\mathrm{standard}}-A_{\mathrm{blank}}}\times C_{\mathrm{standard}}{÷C}_{\mathrm{protein}}$$

The activity of SOD was detected using the xanthine oxidase method, whereby SOD would oxidize hydroxylamine to nitrite and change the color of the chromogenic agent. Concretely, 20 μL of the enzyme-working solution was added to 20 μL of the liver homogenate. We diluted the mixture with 200 μL of the substrate application solution and mixed it thoroughly. Then, we incubated the solution at 37 °C for 20 min and measured the absorbance at 450 nm. The inhibition rate of SOD was calculated using Equation (5). One unit of SOD activity (U) was defined as the amount of enzyme associated with 50% inhibition in the oxidation reaction, as shown in Equation (6).

(5) $$\mathrm{SOD}\;\mathrm{inhibit}\;\mathrm{rate}\;\left(\%\right)=\frac{\left(A_{\mathrm{control}}-A_{\mathrm{control}\;\mathrm{blank}}\right)-\left(A_{\mathrm{test}}-A_{\mathrm{test}\;\mathrm{blank}}\right)}{\left(A_{\mathrm{control}}-A_{\mathrm{control}\;\mathrm{blank}}\right)}\times 100\%$$

(6) $$\mathrm{SOD}\;\mathrm{activity}=\mathrm{SOD}\;\mathrm{inhibite}\;\mathrm{rate}÷50\%\times \mathrm{reaction}\;\mathrm{coefficient}÷C_{\mathrm{protein}}$$

### 2.4. Pesticide Residue Analysis in the Tissues of Mice

A representative 200 mg of liver or 100 mg of kidney, testis, and epididymal fat samples were homogenized individually in 1 mL of acetonitrile containing 1% formic acid for 2 min. The mixture was then centrifuged at 10,000 rpm for 5 min, the supernatant was collected, and then the remaining mixture was extracted one more time. All of the supernatant was combined and degreased using 500 μL of *n*-hexane. The acetonitrile layer was purified with 25 mg of primary secondary amine (PSA) and 25 mg of anhydrous magnesium sulfate sorbents. Following oscillation and centrifugation, the supernatant was filtered through a 0.22 μm filter for LC–MS/MS detection.

### 2.5. LC–MS/MS Analysis

A liquid chromatograph mass spectrometer (LCMS-8045, Shimadzu Company of Japan, Kyoto, Japan) equipped with an Athena C_18_-WP column (3.0 μm × 2.1 mm × 100 mm) was used to detect carbendazim and tebuconazole. The mobile phase was a mixture composed of acetonitrile and water containing 0.1% formic acid (80: 20, *v*/*v*). The sample injection volume was 1 μL. An electrospray ionization (ESI) ion source was operated in the positive mode. The interface voltage was at 4.0 kV. The mass spectrometer was used in the multiple reaction monitoring (MRM) mode. Other mass spectrometry parameters were as follows: 300 °C of ion source temperature, 250 °C of DL tube temperature, and 400 °C of heat block temperature. Nebulizer gas (nitrogen) at 3.0 L/min, heating gas (air) at a flow rate of 10.0 L/min, and drying gas (nitrogen) at 10.0 L/min were employed. Partial instrumental parameters are listed in Table 1.

### 2.6. Analytical Method Validation

The linearity, limit of detection (LOD), limit of quantitation (LOQ), and recovery were tested as method validation for the liver, kidney, testis, and epididymal fat samples. The LOD was defined as the concentration with a signal-to-noise ratio (S/N) of 3, whereas the LOQ was defined as a S/N of 10. The LOQ was established at the lowest spiked concentration of the validation that presented satisfactory precision (RSD ≤ 20%) and recovery (70–110%).

### 2.7. Statistical Analysis

The results are represented as the mean ± standard error of the mean (SEM). Statistical analyses were performed using SPSS 20.0 (IBM Corp., Armonk, NY, USA). The normality of the variables was confirmed by the Shapiro–Wilk test and the homogeneity of variance was determined using Levene’s test. A one-way ANOVA with a post hoc Tukey multiple comparison test was used for the comparison among four groups, and an independent sample t-test was used for the comparison between two groups. *p* < 0.05 was considered statistically significant.

## 3. Results

### 3.1. Method Validation

Recovery and precision were evaluated by spiking samples with the analytes at three concentration levels in triplicate. As shown in Table 2, the recoveries of carbendazim in the liver, kidney, testis, and epididymal fat were in the range of 76.4–93.8% with relative standard deviations (RSDs) ranging from 1.6 to 7.6%. The limit of quantitation (LOQ) of carbendazim in mouse tissues, based on the measured minimum amount in the samples, ranged from 0.5 to 1 μg/kg. The limit of detection (LOD) of carbendazim ranged from 0.15 to 0.3 μg/kg. For tebuconazole, the recoveries in mouse tissues were in the range of 73.9–93.5% with RSDs ranging from 2.6 to 8.9%. The LOQs and LODs of tebuconazole for the mouse tissues were in the range of 0.5–1 μg/kg and 0.15–0.3 μg/kg, respectively. Table 3 shows that the linear calibration curves were obtained over the concentration range of 1–100 μg/kg in the liver and 0.5–50 μg/kg in other tissues of mice for both carbendazim and tebuconazole with correlation coefficients (R^2^) ranging from 0.9937 to 0.9999. To sum up, these results indicated that the method was sufficiently reliable for the carbendazim and tebuconazole analyses with a high precision and accuracy.

### 3.2. Accumulation and Distribution of Carbendazim and Tebuconazole in Mice

To investigate whether carbendazim and tebuconazole would accumulate in the tissues when they were exposed to their acceptable daily intake (ADI) doses for 4 weeks, the concentrations in the liver, kidney, testis, and epididymal fat of the mice were analyzed. The concentrations measuring below the LOQs were regarded as not detected (ND). As shown in Table 4, tebuconazole was mainly observed in the liver, testis, and epididymal fat of mice in the tebuconazole group. The highest concentration of tebuconazole was detected in the epididymal fat with the mean value of 16.84 μg/kg. Lower levels of tebuconazole residue were found in the liver and testis samples, in which the results were under the LOQ level in some samples. In comparison to tebuconazole, carbendazim was not found in the samples when exposed to the ADI dose for 4 weeks.

Because no significant residues were found in some tissues when exposed to the two pesticides at the ADI dose, the mice were exposed to carbendazim and tebuconazole at the 100-fold ADI dose to investigate the distribution character in the tissues. It was found that tebuconazole could be detected in most of the tissue samples when the exposure concentration was increased 100-fold ADI (Table 5). The highest accumulation concentration was found in epididymal fat with the mean value of 28.10 μg/kg. The residual concentrations in the testis and liver were also higher than those in the group exposed to the ADI level. As for carbendazim, there were still virtually no measurable residues in the mice after administration at the 100-fold ADI dose.

### 3.3. Effects of Carbendazim and Tebuconazole on Hepatic Oxidative Stress

**Figure 1:**
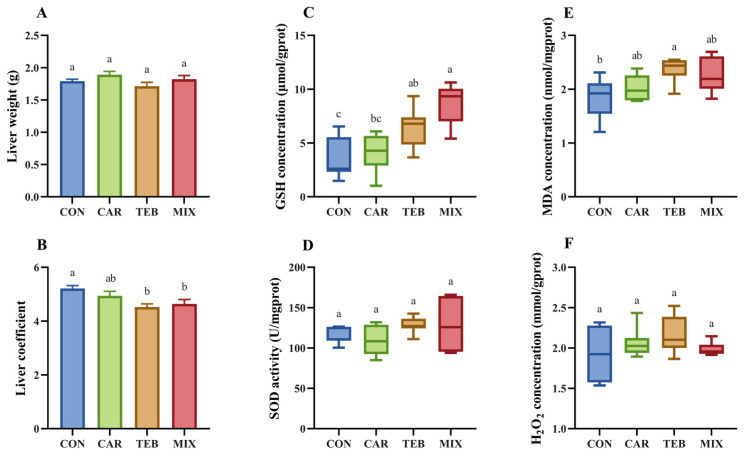
The influence of carbendazim and tebuconazole on liver oxidative stress. (**A**) Liver weight; (**B**) liver coefficient, computed by dividing the weight of the liver by that of the corresponding mouse; (**C**) liver glutathione (GSH) concentration; (**D**) liver superoxide dismutase (SOD) activity; (**E**) liver malondialdehyde (MDA) concentration; (**F**) liver hydrogen peroxide (H_2_O_2_) concentration. Data were expressed as the mean ± SEM (*n* = 7). Different letters (a, b, c) indicate significantly different values according to the one-way ANOVA with Tukey’s test (*p* < 0.05). CON: the control group; CAR: the ADI carbendazim administration group; TEB: the ADI tebuconazole administration group; MIX: the 100-fold ADI of a combination of carbendazim and tebuconazole administration group.

The influence of the two fungicides on the liver was studied by measuring the liver weight, liver coefficient, and hepatic redox homeostasis, and the results are depicted in Figure 1. There was no significant influence on the liver weight after 4 weeks of exposure to tebuconazole at the ADI dose (1.7 g). However, the liver coefficient (4.5%) was notably reduced compared to the control group (5.2%, *p* < 0.05). Carbendazim at the ADI dose showed no effects on both hepatic weight (1.9 g) and the coefficient (4.9%). When raising the exposure dose to 100-fold ADI, the liver weight was still not affected (1.8 g), but the liver coefficient was significantly decreased (4.6%, *p* < 0.01). The result indicated that exposure to low doses of tebuconazole might still have adverse effects on the liver. To explore the effect of low doses of carbendazim and tebuconazole on the hepatic redox homeostasis, the levels of GSH, H_2_O_2_, and MDA, and the activities of SOD were evaluated. At the ADI dose, tebuconazole caused higher levels of GSH (6.4 μmol/gprot, *p* < 0.05), while carbendazim had no significant effect on GSH (4.0 μmol/gprot) compared to the control group (3.4 μmol/gprot) (Figure 1C), indicating that tebuconazole was harmful to the hepatic antioxidant system. As a lipid peroxidation product, the level of MDA was elevated by tebuconazole at ADI (2.4 nmol/mgprot, *p* < 0.05), compared with 1.9 nmol/mgprot in the control group, but not affected by carbendazim (2.0 nmol/mgprot) (Figure 1E). No statistically considerable changes were found in the activities of SOD or in the levels of H_2_O_2,_ among the groups treated with tebuconazole and carbendazim at ADI compared to the control group (Figure 1D,F). When raising the exposure dose 100-fold ADI in the MIX group, the concentration of GSH (8.6 μmol/gprot, *p* < 0.01) was much higher. However, there was still no obvious variation in the activity of SOD, nor in the measured levels of MDA and H_2_O_2_ (Figure 1D–F). These results suggest that carbendazim at its ADI dose might have no appreciable impacts on the hepatic redox homeostasis, whereas tebuconazole administration could induce certain hepatic oxidative stress in mice.

## 4. Discussion

In general, drug transporters expressed on cell membranes mediate the movement of pesticides into and out of cells. Thus, it is possible that pesticides accumulate in mouse tissues. However, few studies have evaluated the distribution and accumulation of carbendazim and tebuconazole in animals at low doses and long term. In this study, the accumulation of carbendazim and tebuconazole in mouse tissues was investigated by LC–MS/MS. Following 4 weeks of individual exposure to carbendazim and tebuconazole at their acceptable daily intake dose, the results indicated that tebuconazole could be accumulated in the organs of mice, especially in the epididymal fat, followed by the testis and liver. The accumulation of tebuconazole in epididymal fat might be associated with its lipid solubility [28,29]. The accumulation of tebuconazole in the testis might be related to the adherent encapsulation of epididymal fat. Previous studies have demonstrated that tebuconazole can induce reproductive toxicity and impair the spermatogenesis of mammals [17,30,31]. These damages might be linked to the accumulation in the testis. Carbendazim was not significantly accumulated in the tissue no matter at the ADI or 100-fold ADI dose. It was speculated that the daily intake of carbendazim by mice might be timely metabolized and excreted. Therefore, the residue in the tissues were lower than the LOQs of the analytical method.

Although studies have reported the adverse effects of oxidative stress induced by carbendazim and tebuconazole [1,19,22,24,32,33,34,35,36], it remained unclear whether they would be harmful when exposed at the ADI dose. In this research, carbendazim at its ADI dose had no effects on the liver, and led to no obvious damage to the hepatic redox homeostasis. On the contrary, exposure to tebuconazole at its ADI dose posed notable increases in the concentration of glutathione (GSH) in the liver. As a redox-active molecule, GSH participates in many antioxidant reactions to the scavenging superoxide in vivo [37,38]. Previous studies showed that tebuconazole at 0.05 mg/L would slightly increase the GSH concentrations, whereas a high dose of 5 mg/L would distinctly decrease the GSH level in zebrafish [39]. The elevated GSH level in this study might be associated with the low exposure dose. Moreover, malondialdehyde (MDA), a lipid peroxidation product of polyunsaturated fatty acids, is always used as an indicator of oxidative stress, and subsequent cellular injury in cells and tissues [40]. Previous studies have reported that tebuconazole can increase MDA in the liver, kidney, and ventricular myocytes in mice and rats [16,41,42]. In this study, the concentration of MDA was also significantly increased by tebuconazole. What is more, a similar increase in the GSH level was observed while no marked change was found in MDA levels in the liver of mice exposed to carbendazim and tebuconazole at the 100-fold ADI dose. The underlying mechanisms of carbendazim and tebuconazole interactions and the potential combined risks remain for further investigation.

## 5. Conclusions

The accumulation and the influence of carbendazim and tebuconazole on the hepatic oxidative stress in mice at low doses in a 4-week period were investigated. Carbendazim had no significant residuals in mice and no effect on the hepatic redox homeostasis at the ADI level. Tebuconazole accumulated primarily in the epididymal fat and induced hepatic oxidative stress at the ADI dose. Under the exposure of the combination of carbendazim and tebuconazole at the 100-fold ADI, a higher concentration of tebuconazole was found in the tissues, especially in the epididymal fat, while no detectable carbendazim was found, and more severe oxidative stress in the liver was induced compared to the groups exposed to carbendazim or tebuconazole at the ADI. However, the mechanism of hepatic oxidative stress that tebuconazole induced was not explored and needs further investigation in the future. The assessments of pesticide on the environment and human health at low doses and long term, especially in terms of a wide range of new toxic endpoints, should be considered.

## Figures and Tables

**Table 1:** The parameters of mass spectrometry of the analytes.

Compounds	Parent Mass (*m*/*z*)	Product Mass (*m*/*z*)	Collision Energy (eV)
Carbendazim	192.10	160.10 *	−18.0
		132.10	−30.0
		105.15	−39.0
Tebuconazole	308.10	70.05 *	−24.0
		125.05	−37.0
		151.05	−26.0

* Quantification ion.

**Table 2:** Average recovery precision of carbendazim and tebuconazole in the samples.

Sample	Concentration (μg/kg)	Carbendazim	Tebuconazole
		Recovery ± RSD * (%)	Recovery ± RSD (%)
Liver	100	86.8 ± 2.4	93.2 ± 2.7
	10	92.2 ± 3.5	91.7 ± 2.6
	1	89.5 ± 5.5	88.9 ± 6.2
Kidney	50	93.8 ± 1.6	92.2 ± 3.6
	5	87.2 ± 6.3	90.7 ± 4.7
	0.5	86.5 ± 5.7	87.6 ± 6.4
Testis	50	91.3 ± 2.6	90.0 ± 3.5
	5	87.9 ± 5.8	84.6 ± 4.9
	0.5	84.3 ± 6.7	79.7 ± 5.5
Epididymal fat	50	90.0 ± 2.1	93.5 ± 2.8
	5	86.4 ± 2.2	89.2 ± 3.1
	0.5	76.4 ± 7.6	73.9 ± 8.9

* RSD, relative standard deviation.

**Table 3:** Linear calibration curves, linearity ranges, and LOQs of carbendazim and tebuconazole in the samples.

Sample		Carbendazim	Tebuconazole
Liver	Linear calibration curve	y = 5861.5x + 10661	y = 7526.2x + 7837.6
	Linearity range (μg/kg)	1–100	1–100
	R^2^ *	0.9997	0.9999
	LOQ (μg/kg)	1	1
Kidney	Linear calibration curve	y = 6785.2x + 8507.2	y = 8335.2x + 11684
	Linearity range (μg/kg)	0.5–50	0.5–50
	R^2^	0.9997	0.9994
	LOQ (μg/kg)	0.5	0.5
Testis	Linear calibration curve	y = 7972.5x + 11211	y = 7906.2x + 8596.5
	Linearity range (μg/kg)	0.5–50	0.5–50
	R^2^	0.9996	0.9978
	LOQ (μg/kg)	0.5	0.5
Epididymal fat	Linear calibration curve	y = 11256x − 10722	y = 8573.1x − 6992.8
	Linearity range (μg/kg)	0.5–50	0.5–50
	R^2^	0.9937	0.9955
	LOQ (μg/kg)	0.5	0.5

* R^2^, correlation coefficient.

**Table 4:** Concentration of carbendazim and tebuconazole in mouse tissues after 4 weeks of administration of the ADI dose.

Sample	Carbendazim Residual Range (μg/kg)	Tebuconazole Residual Range (μg/kg)
Liver	ND *	ND-24.33
Kidney	ND	ND
Testis	ND	ND-15.61
Epididymal fat	ND	12.71–21.98

* ND, not detected.

**Table 5:** Concentration of carbendazim and tebuconazole in mouse tissues after 4 weeks of administration at the 100-fold ADI dose.

Sample	Carbendazim Residual Range (μg/kg)	Tebuconazole Residual Range (μg/kg)
Liver	ND *	ND-20.30
Kidney	ND	ND
Testis	ND	ND-53.97
Epididymal fat	ND	14.93–55.32

* ND, not detected.

## Data Availability

Not applicable.
